# Assessment of Parkinsonian gait in older adults with dementia via human pose tracking in video data

**DOI:** 10.1186/s12984-020-00728-9

**Published:** 2020-07-14

**Authors:** Andrea Sabo, Sina Mehdizadeh, Kimberley-Dale Ng, Andrea Iaboni, Babak Taati

**Affiliations:** 1 KITE, Toronto Rehabilitation Institute, University Health Network, 550 University Avenue, Toronto, ON M5G 2A2 Canada; 2 Institute of Biomaterials and Biomedical Engineering, University of Toronto, 164 College Street. Room 407, Toronto, ON M2S 3G9 Canada; 3 Department of Psychiatry, University of Toronto, 250 College Street, 8th floor, Toronto, ON M5T 1R8 Canada; 4 Centre for Mental Health, University Health Network, 33 Russell Street, Toronto, ON M5S 2S1 Canada; 5 Department of Computer Science, University of Toronto, 10 King’s College Road, Room 3302, Toronto, ON M5S 3G4 Canada

**Keywords:** Parkinsonism, Dementia, Gait, Computer vision, Human pose tracking

## Abstract

**Background:**

Parkinsonism is common in people with dementia, and is associated with neurodegenerative and vascular changes in the brain, or with exposure to antipsychotic or other dopamine antagonist medications. The detection of parkinsonian changes to gait may provide an opportunity to intervene and address reversible causes. In this study, we investigate the use of a vision-based system as an unobtrusive means to assess severity of parkinsonism in gait.

**Methods:**

Videos of walking bouts of natural gait were collected in a specialized dementia unit using a Microsoft Kinect sensor and onboard color camera, and were processed to extract sixteen 3D and eight 2D gait features. Univariate regression to gait quality, as rated on the Unified Parkinson’s Disease Rating Scale (UPDRS) and Simpson-Angus Scale (SAS), was used to identify gait features significantly correlated to these clinical scores for inclusion in multivariate models. Multivariate ordinal logistic regression was subsequently performed and the relative contribution of each gait feature for regression to UPDRS-gait and SAS-gait scores was assessed.

**Results:**

Four hundred one walking bouts from 14 older adults with dementia were included in the analysis. Multivariate ordinal logistic regression models incorporating selected 2D or 3D gait features attained similar accuracies: the UPDRS-gait regression models achieved accuracies of 61.4 and 62.1% for 2D and 3D features, respectively. Similarly, the SAS-gait models achieved accuracies of 47.4 and 48.5% with 2D or 3D gait features, respectively.

**Conclusions:**

Gait features extracted from both 2D and 3D videos are correlated to UPDRS-gait and SAS-gait scores of parkinsonism severity in gait. Vision-based systems have the potential to be used as tools for longitudinal monitoring of parkinsonism in residential settings.

## Background

Older adults with dementia are more likely to develop gait disorders than those without dementia, including the development of parkinsonism [1]. While some of this parkinsonism is associated with neurodegenerative and vascular changes related to the disease, individuals with dementia are also at risk of being prescribed antipsychotic medication and of developing antipsychotic induced parkinsonism (AIP) [2]. The incidence rate of AIP in older adults with dementia has been estimated to be between 30% to over 60% when treated with conventional antipsychotics [2], while high doses of atypical antipsychotics are also associated with similar levels of AIP in older adults with dementia [3]. Deterioration of mobility function is often associated with parkinsonism and can make tasks of daily living more challenging, reducing the independence of affected individuals and increasing the risk of falls and fall-related injuries. However, AIP is reversible with antipsychotic discontinuation, and thus early detection of AIP may help to prevent excess disability.

Currently, parkinsonism in gait is assessed using the gait criterion of the Unified Parkinson’s Disease Rating Scale (UPDRS), and when it is medication-induced, by the gait criterion of the Simpson-Angus Scale (SAS) [4, 5]. However, these assessments require skilled clinicians and are performed infrequently in care settings for people with dementia, thus changes in gait such as those that are adverse effects of antipsychotic medication may be missed. Therefore, there is an opportunity to develop a technology capable of objectively monitoring for parkinsonism in gait in dementia residential care environments.

Previous work on quantifying parkinsonism in gait has focused on patients with Parkinson’s disease (PD) and has not included individuals with dementia who exhibit parkinsonian gait. Studies on quantitative gait analysis in patients with PD have primarily relied on wearable sensors for data collection [6, 7]. However, wearables require physical contact with the patient and suffer from significant challenges with patient adherence in non-clinical settings [8]. Additionally, because locomotion requires coordinated movement of the entire body, multiple sensors are often required to accurately quantify gait features [9]. In contrast, computer vision systems are well-suited for assessment of gait in non-clinical environments as they can be used to monitor movement of the entire body unobtrusively.

Previous studies have used 3D joint locations obtained from Microsoft Kinect sensors to analyze gait of individuals with PD [10–13]. These studies have primarily focused on distinguishing between gait of PD patients and healthy individuals [10], as well as between those who experience freezing of gait and those who do not [11]. A previous study has also successfully used two Kinect sensors positioned in a hallway to distinguish between three stages of PD, ranging from no axial impairment to advanced PD with severe gait disturbances [13].

However, this previous work relies on the Kinect, whose depth sensor can only track individuals at a distance between 0.5 m to 4.5 m, and experiences discontinuities in predicted joint position when a joint is occluded from view of the sensor [14, 15].

Studies using standard video for detection of parkinsonian gait have focused on creating binary masks of the participant through background subtraction [16, 17]. While these approaches are suitable for controlled environments in which there is only one participant and there is visible separation between the participant and background, these techniques would be difficult to deploy in non-clinical setting. Furthermore, the aim of these studies was to identify impairments and presence of Parkinson’s disease rather than distinguishing between varying severities of parkinsonism.

Recent advances in pose tracking algorithms that use deep learning methods have facilitated extraction of joint positions from videos recorded using consumer-grade color cameras [18]. Because these algorithms do not rely on depth data, they are able to predict joint locations over a greater distance, providing opportunity to record more steps of walking. Furthermore, discontinuities in joint coordinates are less frequent as positions of obscured body parts are well inferred by the underlying machine learning model. Joint coordinates extracted through pose tracking on standard videos have already been used successfully in applications of human action recognition [19]. In the field of Parkinson’s disease research, these algorithms have been used in the quantification of dyskinesias [20, 21]; however scoring the *severity* of parkinsonian gait on *clinical scales* has not yet been explored using pose-tracking in video.

The aim of this study is to evaluate whether vision-based systems can be used to identify gait features associated with the severity of parkinsonism in people with dementia, as rated on the UPDRS-gait and SAS-gait scales. It is hypothesized that both 2D and 3D gait features capture clinically relevant aspects of gait and are thus both correlated with parkinsonism severity in gait, rated on the UPDRS-gait and SAS-gait scales.

## Methods

### Data collection

The data used for this investigation was collected as part of a larger prospective observational study conducted at a specialized dementia unit [22]. All patients in the unit had a diagnosis of dementia. Patients capable of independent ambulation over a distance of 20 m were recruited for the study. Consent was obtained from substitute decision makers for all participants. Data collection activities were only performed if the participant also provided assent. The Research Ethics Board of the institute approved the study protocol.

**Fig. 1:**
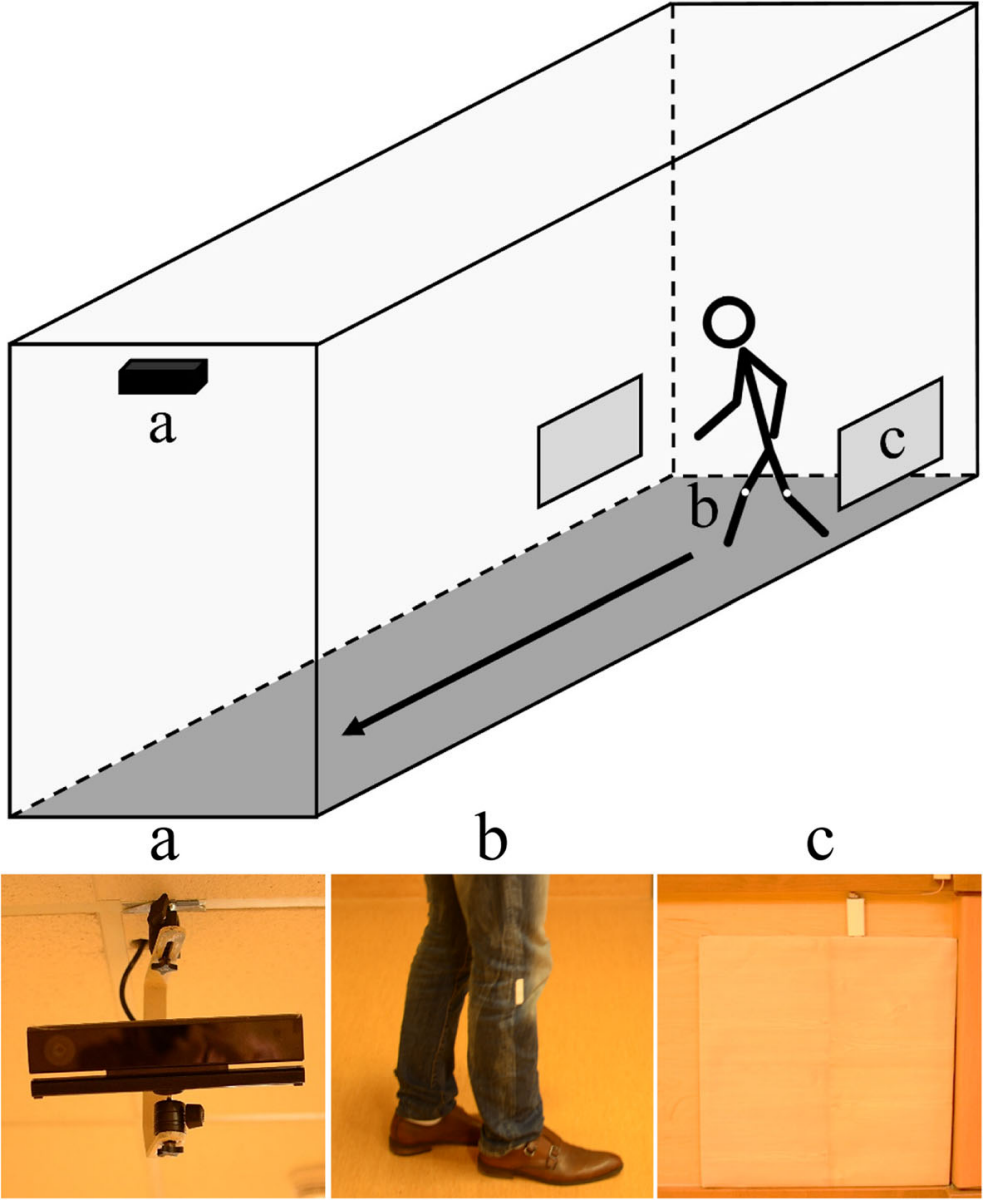
Schematic of data collection equipment in hallway of dementia inpatient unit of a hospital. The Microsoft Kinect (**a**) begins recording when the RFID tags on the participants’ pants (**b**) are detected by the radio-frequency antennae in the walls (**c**)

The data collection system consisted of a Microsoft Kinect for Windows v2 sensor ceiling-mounted in a hallway of the dementia unit. The Kinect was used to simultaneously record standard color video, as well as 3D skeletons using additional data from the onboard depth sensor. A Radio Frequency Identification (RFID) system was used to protect the privacy of non-participating individuals by only collecting data when study participants entered the field of view of the Kinect [23]. The Kinect system was activated when radio-frequency antennae located on the walls of the hallway detected RFID tags attached to the inside of the participants’ pants as they began walking down the hallway. Once triggered, the Kinect recorded 30 s of video of the participant walking down the hallway, towards the camera. A schematic of the data collection system is depicted in Fig. 1. The Kinect records color video at a resolution of 1920 by 1080 pixels at a frequency of 30 Hz and provides the coordinates of 25 body joints in 3D space [14] . Clinical assessment of study participants were also completed as described in [22].

#### Annotation of walks

A set of 14 participants from the larger study were purposively selected for inclusion in this study, such that the sample included a range of parkinsonian gait characteristics. Data was collected over the course of several weeks for each participant who were undergoing changes in treatment with antipsychotic medication; so it was possible for the person’s gait to change from one recording to another. When multiple walking bouts of a participant were captured by the data collection system on a single day, one of the walking bouts for that day was randomly selected. This was done to incorporate longitudinal data collected over the course of several weeks, while also avoiding biasing the dataset with many walks collected on the same day, as walks collected in close succession are postulated to be of similar quality, and therefore have the same clinical scores.

Each selected walking bout was scored on the gait criterion of the UPDRS and SAS scales by an expert annotator. Both the UPDRS and SAS use an integer scale from 0 to 4 to evaluate gait, where a higher score indicates the presence of gait characteristics consistent with more severe parkinsonism.

### Calculation of gait parameters

Gait parameters were computed from the recorded gait sequences in two ways: using the 3D joint coordinates extracted using the Kinect software development kit (SDK), and by directly processing the recorded color videos via the OpenPose library to obtain 2D joint (pixel) coordinates [24].

Gait parameters computed from the 3D joint coordinates include spatiotemporal, variability, symmetry, and mechanical stability measures of gait. A complete list of the 3D features investigated in this study is presented in Table 3. A detailed methodology of how these were computed and validated is presented in previous work [22, 23, 25, 26].

Furthermore, gait parameters were also calculated directly from the videos recorded using the color camera of the Kinect. The first step of this process involved extracting 2D keypoints corresponding to the joint locations of the individuals in the scene. A TensorFlow implementation of the OpenPose library, pretrained on the MPII dataset was used for keypoint extraction [24]. Joint coordinates were extracted in this manner for each frame of the video, allowing the trajectory of movement to be tracked during the walking bout. These 2D keypoints were then used to compute gait parameters such as cadence, average and minimum margin of stability (MOS) per step, average step width, coefficient of variation (CV) of step width and time, the symmetry index (SI) of the step times, as well as the number of steps in the walking bout [27].

To calculate these gait features, the steps in each walking bout were first identified. This was done by differentiating the vertical position of the ankle keypoints with respect to time, and as proposed in [28], denoting foot strikes when this velocity signal passed 35% of the peak value for each cycle. After identifying foot strikes, each step of the walking bout could be isolated allowing the cadence, average step width, CV of step width and step time, SI of step times, and the number of steps in the bout to be calculated. To compute the average and minimum and average MOS per step, the extrapolated centre of mass was first estimated by incorporating a normalized measure of velocity to the centre of the left and right hip keypoints. The difference between the extrapolated centre of mass and the ankle of the stance foot was calculated for each frame. The average and minimum MOS gait features were then estimated by averaging the magnitude of all differences, or only the minimum difference per step, respectively. A detailed description of the calculation of 2D gait features is presented in [27, 29].

Note that for the 2D feature set, it is not possible to compute gait features that rely on distance measures in the depth (anterior-posterior) direction (such as step length or velocity) using the video stream. However, to normalize for the distance of the participant with respect to the camera over the course of a walking bout, all horizontal and vertical distances (in pixels) computed using the 2D keypoints were divided by the distance between the left and right hip keypoints in that frame.

Overall, 16 gait parameters were computed from the 3D joint keypoints, and 8 gait parameters were computed from the 2D joint keypoints.

### Statistical analysis

During preliminary analysis, univariate ordinal logistic regression was performed between each gait parameter and the dependent variable (UPDRS-gait or SAS-gait score). The threshold for statistical significance was set at *p* < 0.05. The goal of this univariate analysis was to select gait parameters to include in the subsequent multivariate regression model. Thus, Bonferroni correction was not applied to avoid prematurely discarding gait features that were truly correlated with the dependent variables.

A multivariate analysis was then performed to evaluate the relative importance of the gait features selected in the univariate analysis for regression to UPDRS-gait and SAS-gait scores. The multivariate models contained the demographic features of age and sex, as well as all gait features that were identified as being significantly correlated to the dependent variable in the univariate analysis. To avoid including gait features that are strongly correlated with each other, Pearson correlation coefficients, **r**, between all gait features were computed. When the magnitude of the correlation coefficient was greater than 0.5, only the gait feature with the larger magnitude correlation to the dependent variable was retained for inclusion in the multivariate model. Therefore, the final multivariate model included the demographic features of age and sex, as well as all of the gait parameters that were significantly correlated with the dependent variable (either UPDRS-gait or SAS-gait score), but not strongly correlated with each other. This statistical analysis was repeated twice, analyzing the set of gait features computed from the 2D and 3D joint locations separately.

## Results

A total of 401 walking bouts from 14 participants (8 male, mean age 76.2 ± 8.7 years) were analyzed. Table 1 presents the clinical characteristics of the participants.

**Table 1:** Clinical characteristics of study participants

		**Study Participants** **(*n*** **= 14)** **Mean ± SD**
Age		76.2 ± 8.7
Sex (% male)		57.1
Number of Walks		28.6 ± 11.8
Total Severe Impairment Battery (SIB) Score		27.9 ± 13.1
Total Neuropsychiatric Inventory (NPI) Score		46.3 ± 19.5
Total Katz Index of Independence in Activities of Daily Living Score		2.3 ± 1.3
Total Tinetti POMA Balance Score		9.9 ± 3.1
Total Tinetti POMA Gait Score		8.4 ± 2.8
Antipsychotic Medication Use	Study Participants Prescribed Medication (*n* = 14)	**Daily Dose (mg)** **Mean ± SD**
Risperidone	3 (21.4%)	0.5 ± 0.4
Quetiapine	11 (78.6%)	64.2 ± 61.8
Clozapine	2 (14.3%)	32.4 ± 7.2
Loxapine	5 (35.7%)	5.5 ± 1.1
Olanzapine	2 (14.3%)	1.9 ± 0.9
Nozinan	2 (14.3%)	12.8 ± 3.8

All 401 bouts had associated Kinect data, however color video was only available for 364 bouts. In 19 bouts, the participant could not be tracked by the OpenPose algorithm, and 96 walks were too short (less than 3 tracked steps), leaving 249 bouts for which 2D gait features were computed, and 398 bouts for which 3D gait features could be calculated. The walks for which 2D gait features were calculated are a subset of the walks for which 3D gait features were computed. However, the mean number of steps per walk captured by the 2D features was 6.9, whereas the 3D gait features captured 5.8 steps per walk. Table 2 presents the distribution of walks for UPDRS-gait and SAS-gait scores for the 2D and 3D gait feature datasets. A breakdown of the distribution of scores and walks for each participant is included in Table A and Table B in Additional file 1.

**Table 2:** Distribution of UPDRS-gait and SAS-gait scores for participants’ walks

		Number of walks			
		2D gait feature dataset		3D gait feature dataset	
		UPDRS-gait	SAS-gait	UPDRS-gait	SAS-gait
Score	0	41	42	90	77
	1	76	76	129	136
	2	132	91	179	136
	3	0	40	0	49
	Total	249	249	398	398

### Univariate analysis

Table 3 presents the regression coefficients and *p*-values for univariate regression to UPDRS-gait and SAS-gait scores using 2D and 3D gait features, respectively.

**Table 3:** Regression coefficients and *p*-values for univariate regression to UPDRS-gait and SAS-gait scores with 2D and 3D gait features

		UPDRS-gait		SAS-gait	
		Regression Coefficient	*p*-value	Regression Coefficient	*p*-value
2D Features	steps of walk	0.14	< 0.001**	0.086	0.0039**
	cadence (steps/min)	0.021	0.0024**	0.023	< 0.001**
	symmetry index (SI) of step time	−0.011	0.0035**	− 0.0090	0.0093**
	coefficient of variation (CV) of step time	−1.0	0.039*	−1.2	0.015*
	average step width	−0.77	0.19	0.0035	0.99
	average margin of stability (MOS)	1.0	0.21	2.2	0.0047**
	average minimum MOS	1.4	0.22	2.8	0.010*
	CV step width	−0.27	0.23	−0.21	0.40
3D Features	walking speed (m/s)	−7.5	< 0.001**	−6.0	< 0.001**
	step length (m)	−10.	< 0.001**	−8.3	< 0.001**
	step width symmetry angle (degrees)	−0.075	< 0.001**	− 0.073	< 0.001**
	root mean square (RMS) of sacrum medio-lateral (ML) velocity	−5.0	< 0.001**	−3.3	0.015*
	step length symmetry angle (degrees)	0.054	< 0.001**	0.050	0.0012**
	average MOS (mm)	−10.	0.0013**	−8.1	0.0071**
	CV step width	−1.5	0.0018**	−1.4	0.0018**
	step width (m)	−4.2	0.047*	−4.3	0.034*
	minimum MOS (mm)	−9.4	0.13	−5.3	0.38
	step time symmetry angle (degrees)	−0.020	0.21	−0.031	0.045*
	CV step length	0.50	0.25	0.19	0.66
	standard deviation (SD) sacrum ML	−1.2	0.57	−3.4	0.11
	cadence (steps/min)	−0.0019	0.63	−0.0024	0.53
	step time (s)	0.27	0.74	0.28	0.71
	CV step time	0.10	0.82	−0.24	0.59
	range of motion (ROM) sacrum ML	0.018	0.98	−0.52	0.36

*significant at *p* < 0.05, **significant at *p* < 0.01

The 2D gait features that were significantly correlated to UPDRS-gait score were the number of steps in the walking bout, cadence, and the symmetry index and coefficient of variation of step time. The 2D gait features correlated to SAS-gait were the same as those for UPDRS-gait, with the addition of the average margin of stability (MOS). The 3D gait features that were correlated to UPDRS-gait were: walking speed, symmetry of step width and length, root mean square (RMS) of medio-lateral velocity of the sacrum, average MOS, and step width. The same 3D gait features were correlated with SAS-gait scores, with the addition of step time symmetry.

### Multivariate analysis

Table 4 presents the regression coefficients and intercepts of the ordinal logistic regression models to UPDRS-gait and SAS-gait after including the 2D gait features selected through the univariate analysis. The smaller the *p*-value for a particular gait feature, the higher the probability that including that gait feature significantly improved the regression model to the clinical variable. When considered individually, no gait features significantly (*p* < 0.05) improved regression to UPDRS-gait score. Conversely, for the regression model to SAS-gait, the cadence, coefficient of variation (CV) of step time, and average MOS all significantly improved the model (*p* < 0.05) when considered individually.

**Table 4:** Regression coefficients, standard error, t-values, and *p*-values for multivariate regression models to UPDRS-gait and SAS-gait with 2D gait features

		UPDRS-gait				SAS-gait			
		Regression Coefficient	Std. Error	t-value	*p*-value	Regression Coefficient	Std. Error	t-value	*p*-value
Coefficients	age	0.11	0.018	6.0	< 0.001**	0.071	0.016	4.4	< 0.001**
	sex (male)	1.7	0.29	5.8	< 0.001**	2.2	0.29	7.6	< 0.001**
	steps of walk	0.024	0.044	0.55	0.58	0.0035	0.034	0.10	0.92
	cadence (steps/min)	0.0089	0.0075	1.2	0.23	0.024	0.0079	3.1	0.0019**
	SI step time	−0.0061	0.0047	−1.3	0.20	−0.0034	0.0044	−0.76	0.44
	CV step time	−1.1	0.62	−1.8	0.080	−1.6	0.62	−2.6	0.011*
	average MOS	N/A	N/A	N/A	N/A	3.2	0.85	3.7	< 0.001**
Intercepts	0|1	7.4	1.3	5.8	< 0.001**	7.5	1.3	5.7	< 0.001**
	1|2	9.5	1.4	7.0	< 0.001**	9.8	1.4	6.9	< 0.001**
	2|3	29	1.4	22	< 0.001**	12	1.5	8.2	< 0.001**
	3|4	30.	1.4	22	< 0.001**	43	1.5	29	< 0.001**

*significant at *p* < 0.05, **significant at *p* < 0.01

A similar multivariate regression model was developed for using the 3D gait features selected during the univariate analysis. Table 5 presents the coefficients and intercepts of the ordinal logistic regression models to UPDRS-gait and SAS-gait with the inclusion of the selected 3D gait features. For both regression models using 3D features, the walking speed, and the step width and length symmetry angles significantly improved regression to UPDRS-gait and SAS-gait when considered individually. Additionally, the RMS of medio-lateral velocity of the sacrum also significantly improved regression to UPDRS-gait, whereas the step width and step time symmetry angle significantly improved the SAS-gait model with 3D features.

**Table 5:** Regression coefficients, standard error, t-values, and p-values for multivariate regression models to UPDRS-gait and SAS-gait with 3D gait features

		UPDRS-gait				SAS-gait			
		Regression Coefficient	Std. Error	t-value	*p*-value	Regression Coefficient	Std. Error	t-value	*p*-value
Coefficients	age	0.098	0.015	6.4	< 0.001**	0.068	0.014	5.0	< 0.001**
	sex (male)	1.6	0.24	6.6	< 0.001**	2.2	0.24	9.0	< 0.001**
	walking speed (m/s)	−5.7	0.96	−5.9	< 0.001**	−3.2	0.81	−3.9	< 0.001**
	step width symmetry angle (degrees)	−0.065	0.019	−3.4	< 0.001**	−0.070	0.018	−3.9	< 0.001**
	RMS sacrum ML velocity	−8.9	2.0	−4.5	< 0.001**	−2.6	1.9	−1.4	0.17
	step length symmetry angle (degrees)	0.074	0.022	3.4	< 0.001**	0.11	0.021	5.4	< 0.001**
	average MOS (mm)	−7.7	5.0	−1.6	0.12	−7.4	4.4	−1.7	0.093
	step width (m)	−3.5	3.2	−1.1	0.27	−9.4	2.9	−3.2	0.0014**
	step time symmetry angle (degrees)	N/A	N/A	N/A	N/A	−0.13	0.021	−6.0	< 0.001**
Intercepts	0|1	0.65	1.5	0.42	0.68	−0.89	1.4	−0.62	0.54
	1|2	3.0	1.6	1.9	0.052	1.7	1.5	1.1	0.26
	2|3	49	1.6	31	< 0.001**	4.3	1.5	3.0	0.0032**
	3|4	50.	1.6	32	< 0.001**	3500	1.5	2400	< 0.001**

*significant at *p* < 0.05, **significant at *p* < 0.01

**Fig. 2:**
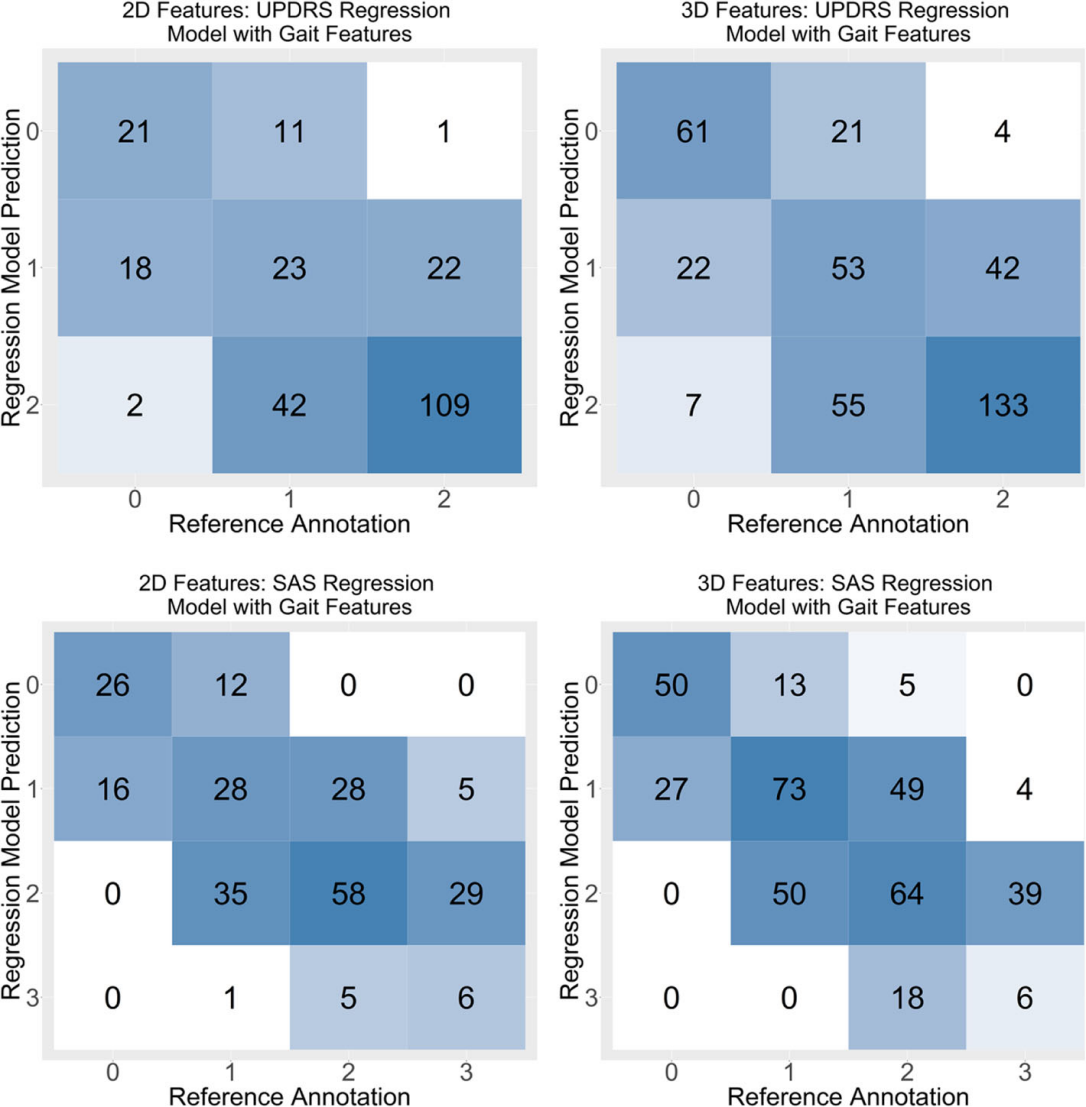
Confusion matrices for regression to final multivariate regression models to UPDRS-gait (top) and SAS-gait (bottom) clinical scores using 2D (left) and 3D (right) gait features

Figure 2 presents the confusion matrices for the final multivariate regression models to UPDRS-gait and SAS-gait using 2D and 3D gait features.

The accuracy of the multivariate regression models by participant is presented in Table 6.

**Table 6:** Final multivariate model accuracies by clinical score and feature set, per participant

Participant	2D gait features			3D gait features		
	UPDRS-gait accuracy	SAS-gait accuracy	Percent of walks	UPDRS-gait accuracy	SAS-gait accuracy	Percent of walks
1	0.0%	0.0%	0.4%	30.8%	15.4%	3.3%
2	100.0%	50.0%	3.2%	100.0%	61.1%	4.5%
3	36.4%	45.5%	4.4%	40.9%	36.4%	5.5%
4	86.4%	45.5%	17.7%	64.0%	48.0%	12.6%
5	53.3%	66.7%	6.0%	32.1%	75.0%	7.0%
6	100.0%	76.2%	8.4%	100.0%	47.6%	5.3%
7	60.0%	48.0%	10.0%	69.2%	56.4%	9.8%
8	65.1%	41.9%	17.3%	64.8%	44.4%	13.6%
9	59.1%	27.3%	8.8%	83.3%	13.3%	7.5%
10	28.6%	57.1%	5.6%	47.6%	38.1%	5.3%
11	26.7%	46.7%	6.0%	57.7%	50.0%	6.5%
12	46.2%	53.8%	5.2%	87.1%	80.6%	7.8%
13	30.8%	23.1%	5.2%	40.0%	40.0%	6.3%
14	0.0%	50.0%	1.6%	25.0%	55.0%	5.0%
All	61.4%	47.4%	100.0%	62.1%	48.5%	100.0%

## Discussion

By analyzing natural walking bouts collected from 14 patients over the course of several weeks, this study has identified the gait features that are significantly associated with parkinsonism severity in gait of individuals with dementia, as rated on the UPDRS and SAS scales. Both 2D and 3D gait features capture clinically relevant data that can be used to quantify the severity of parkinsonism in natural gait, so there is an opportunity to use vision-based systems for longitudinal assessment of parkinsonian gait in residential care settings.

Spatiotemporal, variability, and symmetry measures of gait extracted from 2D and 3D video data were found to be significantly associated with UPDRS-gait and SAS-gait clinical measures. These features are consistent with previous studies where marker-based vision systems were used to quantify differences in gait of healthy individuals and those with PD. In particular, decreased stride length and gait velocity, and increased bilateral asymmetry have been found to be significantly associated with parkinsonian gait [30–32]. A limitation of this previous work is that the imaging systems used rely on multiple cameras and body-worn markers, and therefore have limited applicability for monitoring of day-to-day walking outside of laboratory or clinical environments [30, 31]. Our study is the first to validate that these changes in gait are also measurable with increasing severity of parkinsonism in individuals with dementia.

Furthermore, while previous work has demonstrated the feasibility of using gait features extracted using 3D Kinect skeletons to classify parkinsonian gait into three levels of severity [13], to the best of the authors’ knowledge, our work is the first to demonstrate that pose-tracking algorithms on standard color video can be used to estimate 2D gait features that similarly capture characteristics of parkinsonism severity in gait.

The UPDRS-gait regression models incorporating 2D or 3D gait features achieved similar average final accuracies (61.4% vs. 62.1% for 2D and 3D features, respectively). Likewise, the SAS-gait models achieved final accuracies of 47.4 and 48.5% with 2D or 3D gait features. As seen in Table 6, the accuracy of the models differs greatly for each participant. This suggests that while there are general correlations between gait features and parkinsonism when considering the group of all participants, there is also large variation in gait between participants. From Table 6, it can also be observed that walks from participants 4 and 8 represent a higher proportion of all walks in the dataset, so the models are more influenced by these participants. However, the average accuracy of the models when considering only participants 4 and 8; and when considering all 14 participants is similar (within 2.5%) for all except the 2D UPDRS-gait model, suggesting that the models are not overfitting to the two participants with more walks. For the UPDRS-gait model with 2D gait features, the model achieves a higher accuracy on participant 4 (86.4%), while the accuracy on walks for participant 8 is 65.1%, similar to the overall model accuracy of 61.4%.

As observed qualitatively in the confusion matrices in Fig. 2, when there was a discrepancy between the clinician annotation and the model prediction, the difference between predicted UPDRS-gait or SAS-gait score and the reference annotation was 1 category for the majority of walking bouts. This is consistent with the nature of these rating scales, whereby a continuous range of symptoms are discretized and scored on integer scales from 0 and 4. When clinicians make decisions about what score to assign to a particular walking bout, they are usually deciding between two adjacent scores on the scale. A similar pattern is seen in the UPDRS-gait and SAS-gait scores predicted by the regression models, demonstrating that while the overall fit of the models is consistent with clinician annotations, there is some ambiguity between adjacent scores on the scales. Similar ambiguity between adjacent scores on the rating scales is also present for expert annotators, with studies on the reliability of the UPDRS scale reporting that the intraclass correlation coefficient for the gait item is between 0.746 and 0.90 when assessed by multiple clinicians [33, 34]. While clinician ratings are currently the “gold standard,” there is an opportunity, using large video datasets of parkinsonian and normal gait, to develop deep learning models capable of recognizing or categorizing parkinsonian gait, and providing objectively calculated severity scores.

We have also demonstrated that both the Kinect sensor and onboard color camera provide similar results, suggesting that consumer-grade video cameras are sufficient to capture clinically significant characteristics of parkinsonian gait. Given the similar accuracy of the models with 2D and 3D gait features, the advantage of using 2D gait features is that they can be computed from video collected with any standard consumer-grade camera, whereas the 3D gait features require specialized imaging systems with depth sensors such as the Microsoft Kinect. For low-cost or mobile applications, or in instances where the individual is more than 4.5 m from the camera, 2D gait features are better suited to quantify severity of parkinsonism in gait. Conversely, for applications where it is possible to use larger and more sophisticated imaging systems, 3D features may be better suited as they can also capture spatiotemporal gait features that rely on depth data such as step length and walking speed.

## Conclusions

In this study, we demonstrated that both 2D and 3D gait features calculated from video are correlated to clinical measures of parkinsonism severity in gait, as rated on the UPDRS and SAS scales. These findings suggest that both 2D and 3D vision systems have applications in longitudinal monitoring of parkinsonism severity in residential settings.

The strength of this study is that we were able to analyze natural walking bouts of individuals with dementia and compare the 2D and 3D gait features associated with parkinsonism. Because both 2D and 3D gait features were calculated for most walking bouts, direct comparison between the association of 2D and 3D gait features and clinical scores was possible. A limitation of this study is that the regression models developed were not well suited for prediction as they could only identify linear relationships between the gait features and clinical scores. Future work will extend the results obtained in this study and focus on developing predictive models for scoring severity of parkinsonism in gait using timeseries data of joint coordinates, without relying on the explicit computation of gait features. It is hypothesized that developing machine learning models that do not rely on handcrafted features will be able to better capture latent structure in the underlying data and thus learn to predict UPDRS-gait and SAS-gait scores more accurately.

## Supplementary information

**Additional file 1: Table A.** Distribution of UPDRS-gait and SAS-gait scores by participant for walks with 2D gait features. **Table B.** Distribution of UPDRS-gait and SAS-gait scores by participant for walks with 3D gait features.

## Data Availability

The datasets generated and/or analysed during the current study are not publicly available due to the vulnerability of the population (ie. individuals with dementia) and the personal nature of video recordings captured in an inpatient environment, but preprocessed and deidentified gait features used in the analysis are available from the corresponding author on reasonable request.

## Abbreviations

2D: Two-dimensional
3D: Three-dimensional
AIP: Antipsychotic induced parkinsonism
CV: Coefficient of variation
ML: Medio-lateral
MOS: Margin of stability
PD: Parkinson’s disease
RFID: Radio Frequency Identification
RMS: Root mean square
ROM: Range of motion
SAS: Simpson-Angus Scale
SD: Standard deviation
SI: Symmetry index
SDK: Software development kit
UPDRS: Unified Parkinson’s Disease Rating Scale
