# Supplementary material for: Assessment of Parkinsonian gait in older adults with dementia via human pose tracking in video data
Source: J Neuroeng Rehabil. 2020 Jul 14;17:97. doi: 10.1186/s12984-020-00728-9 (PMC7362631; doi:10.1186/s12984-020-00728-9)
Supplement: Supplementary file 1 — Additional file 1: Table A. Distribution of UPDRS-gait and SAS-gait scores by participant for walks with 2D gait features. Table B. Distribution of UPDRS-gait and SAS-gait scores by participant for walks with 3D gait features. [file 12984_2020_728_MOESM1_ESM.docx]

Table A: Distribution of UPDRS-gait and SAS-gait scores by participant for walks with 2D gait features

| **Participant** | **2D Gait Features** | | | | | | | | | |
| --- | --- | --- | --- | --- | --- | --- | --- | --- | --- | --- |
|  | **UPDRS-gait score** | | | | **SAS-gait score** | | | | | |
|  | **0** | **1** | **2** | **All** | **0** | **1** | **2** | **3** | **All** |  |
| 1 | 0 | 1 | 0 | 1 | 0 | 0 | 1 | 0 | 1 |  |
| 2 | 0 | 0 | 8 | 8 | 0 | 0 | 5 | 3 | 8 |  |
| 3 | 0 | 4 | 7 | 11 | 0 | 5 | 5 | 1 | 11 |  |
| 4 | 0 | 8 | 36 | 44 | 0 | 9 | 32 | 3 | 44 |  |
| 5 | 0 | 9 | 6 | 15 | 0 | 14 | 1 | 0 | 15 |  |
| 6 | 0 | 0 | 21 | 21 | 0 | 0 | 18 | 3 | 21 |  |
| 7 | 11 | 14 | 0 | 25 | 15 | 9 | 1 | 0 | 25 |  |
| 8 | 0 | 14 | 29 | 43 | 0 | 13 | 21 | 9 | 43 |  |
| 9 | 0 | 2 | 20 | 22 | 0 | 0 | 2 | 20 | 22 |  |
| 10 | 14 | 0 | 0 | 14 | 14 | 0 | 0 | 0 | 14 |  |
| 11 | 0 | 14 | 1 | 15 | 0 | 13 | 2 | 0 | 15 |  |
| 12 | 13 | 0 | 0 | 13 | 13 | 0 | 0 | 0 | 13 |  |
| 13 | 0 | 9 | 4 | 13 | 0 | 9 | 3 | 1 | 13 |  |
| 14 | 3 | 1 | 0 | 4 | 0 | 4 | 0 | 0 | 4 |  |
| **All** | **41** | **76** | **132** | **249** | **42** | **76** | **91** | **40** | **249** |  |

Table B: Distribution of UPDRS-gait and SAS-gait scores by participant for walks with 3D gait features

| **Participant** | **3D Gait Features** | | | | | | | | | |
| --- | --- | --- | --- | --- | --- | --- | --- | --- | --- | --- |
|  | **UPDRS-gait score** | | | | **SAS-gait score** | | | | | |
|  | **0** | **1** | **2** | **All** | **0** | **1** | **2** | **3** | **All** |  |
| 1 | 0 | 12 | 1 | 13 | 0 | 0 | 13 | 0 | 13 |  |
| 2 | 0 | 0 | 18 | 18 | 0 | 0 | 14 | 4 | 18 |  |
| 3 | 0 | 8 | 14 | 22 | 0 | 10 | 11 | 1 | 22 |  |
| 4 | 0 | 11 | 39 | 50 | 0 | 14 | 33 | 3 | 50 |  |
| 5 | 0 | 17 | 11 | 28 | 0 | 24 | 4 | 0 | 28 |  |
| 6 | 0 | 0 | 21 | 21 | 0 | 0 | 18 | 3 | 21 |  |
| 7 | 22 | 17 | 0 | 39 | 25 | 13 | 1 | 0 | 39 |  |
| 8 | 0 | 17 | 37 | 54 | 0 | 15 | 28 | 11 | 54 |  |
| 9 | 0 | 2 | 28 | 30 | 0 | 0 | 4 | 26 | 30 |  |
| 10 | 21 | 0 | 0 | 21 | 20 | 1 | 0 | 0 | 21 |  |
| 11 | 0 | 25 | 1 | 26 | 0 | 24 | 2 | 0 | 26 |  |
| 12 | 31 | 0 | 0 | 31 | 31 | 0 | 0 | 0 | 31 |  |
| 13 | 0 | 16 | 9 | 25 | 0 | 16 | 8 | 1 | 25 |  |
| 14 | 16 | 4 | 0 | 20 | 1 | 19 | 0 | 0 | 20 |  |
| **All** | **90** | **129** | **179** | **398** | **77** | **136** | **136** | **49** | **398** |  |
